# Phenazine oxidation by a distal electrode modulates biofilm morphogenesis

**DOI:** 10.1016/j.bioflm.2020.100025

**Published:** 2020-05-13

**Authors:** William Cole Cornell, Yihan Zhang, Anastasia Bendebury, Andreas J.W. Hartel, Kenneth L. Shepard, Lars E.P. Dietrich

**Affiliations:** a Department of Biological Sciences, Columbia University, New York, NY, 10027, USA; b Department of Electrical Engineering, Columbia University, New York, NY, 10027, USA

**Keywords:** *Pseudomonas aeruginosa*, Extracellular electron transfer, Biofilm matrix, Phenazines

## Abstract

Microbes living in biofilms, dense assemblages of cells, experience limitation for resources such as oxygen when cellular consumption outpaces diffusion. The pathogenic bacterium *Pseudomonas aeruginosa* has strategies for coping with hypoxia that support cellular redox balancing in biofilms; these include (1) increasing access to oxygen by forming wrinkles in the biofilm surface and (2) electrochemically reducing endogenous compounds called phenazines, which can shuttle electrons to oxidants available at a distance. Phenazine-mediated extracellular electron transfer (EET) has been shown to support survival for *P. aeruginosa* cells in anoxic liquid cultures, but the physiological relevance of EET over a distance for *P. aeruginosa* biofilms has remained unconfirmed. Here, we use a custom-built electrochemistry setup to show that phenazine-mediated electron transfer at a distance inhibits wrinkle formation in *P. aeruginosa* biofilms. This result demonstrates that phenazine-dependent EET to a distal oxidant affects biofilm morphogenesis.

## Introduction

Microbes commonly grow as biofilms, assemblages of cells that adhere to each other and to objects using a self-produced polysaccharide matrix. Cells in biofilms are subjected to resource gradients due to inhibited diffusion and localized metabolic activities [1]. The success of biofilm-forming microbes, therefore, depends on their ability to face the challenge of resource limitation. Because biofilm growth affects many aspects of human health, it is important to understand the adaptive mechanisms that support this microbial lifestyle [[2], [3], [4], [5]].

**Fig. 1:**
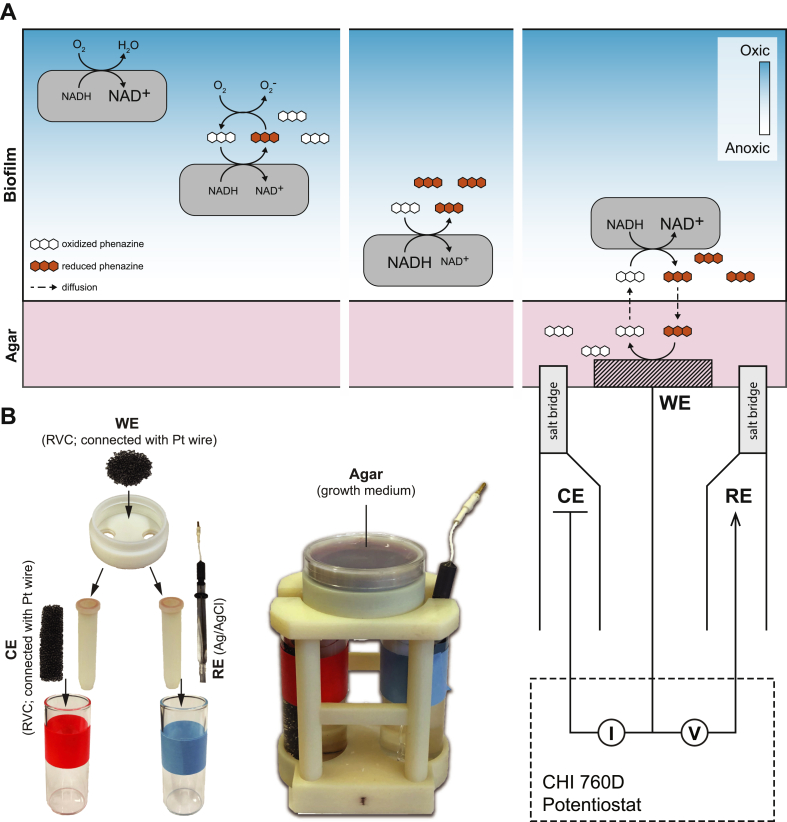
Roles of phenazines as electron acceptors for *P. aeruginosa* biofilm metabolism and experimental setup for testing the model. **(A) Left panel:** Reduction of O_2_ and phenazines by cells in a colony biofilm. Reduced phenazines can transfer electrons to O_2_ present in the upper portion of the biofilm, thereby supporting efficient redox balancing in cells that cannot directly access O_2_. **Middle panel:** With increasing distance from O_2_, reoxidation of phenazines becomes less efficient and cells accumulate the intracellular electron donor NADH. The more reducing cytoplasm promotes the production of extracellular matrix and colony wrinkling (not shown). **Right panel:** A poised-potential electrode at the biofilm base allows for reoxidation of phenazines and redox homeostasis in cells deep in the biofilm. The use of an experimental setup with a distal, non-diffusible oxidant allows us to test whether phenazine-dependent electron transfer affects biofilm physiology. The potentiostat sets the voltage difference between the working electrode (WE) and the reference electrode (RE) and monitors the current passing through the WE to the counter electrode (CE). **(B)** Photographs of the experimental system, shown as individual components (left) and assembled (right).

To study adaptations to biofilm growth, we use a colony morphology model of the pathogenic bacterium *Pseudomonas aeruginosa*. In this model, two main oxidants act as metabolic electron acceptors (Fig. 1A) [6]: (i) oxygen (O_2_), a respiratory substrate, and (ii) phenazines, endogenously produced small molecules that can shuttle electrons to O_2_, iron oxides or a poised-potential electrode [7,8]. O_2_ or phenazine reduction supports cellular redox homeostasis, a condition that is required for optimal metabolic functioning and survival. Oxidant availability influences the onset and/or degree of matrix production during *P. aeruginosa* biofilm development [9,10]. In conditions of oxidant limitation, matrix production promotes vertical structure formation, i.e. “wrinkling”, which increases the surface area of the biofilm and therefore access to O_2_ for the cells within [6,9,11]. Polysaccharide staining has shown that matrix production occurs at a specific, median depth in the biofilm and is enhanced in mutants that are defective in O_2_ respiration or phenazine biosynthesis [11,12]. Microelectrode measurements of biofilms reveal that with increasing distance from the surface of the biofilm, O_2_ concentrations fall below the limit of detection (0.3 ​μM) and the extracellular phenazine pool becomes more reduced [9,12]. Together, these observations suggest that oxidant availability is a cue that inhibits matrix production in the median biofilm subzone.

Consistent with their favorable redox potentials and redox-cycling properties, phenazines promote survival in anaerobic *P. aeruginosa* cell suspensions when an electrode is provided to support phenazine oxidation [8]. This observation led to the model that phenazines could serve a similar function in biofilms by shuttling electrons from cells inhabiting O_2_-limited subzones to the more O_2_-replete zone near the surface. While our previous studies have demonstrated that phenazines facilitate redox balancing and promote metabolic activity at depth in biofilms [9,13], an open question that has remained is whether a distal, non-diffusible oxidant could affect biofilm physiology by shifting the oxidation state of the phenazine pool. Because O_2_ can oxidize phenazines and can diffuse into the upper portion of the biofilm, it has been technically challenging to demonstrate electron shuttling via phenazine diffusion per se. To address this problem, we engineered a device that supports bulk electrolysis in the growth medium used in our standard colony morphology assay (Fig. 1B). Here, we describe the use of this device to test whether *P. aeruginosa* biofilms catalyze phenazine-mediated electron transfer at a distance and whether this activity inhibits biofilm wrinkle formation.

## Results and Discussion

To allow us to test the effect of electron transfer over a distance on biofilm development, we designed an apparatus that mimics our standard biofilm growth conditions but that also provides compatibility with external electrochemical stimulation (Fig. 1B). Biofilms were grown within this device in a vessel with dimensions similar to a 35 ​× ​10 ​mm Petri dish (Falcon). A reticulated vitreous carbon (RVC) foam electrode was chosen as the working electrode for its large area to volume ratio and was fully submerged in the agar-solidified growth medium. Two openings at the bottom of the dish allowed for electrical salt bridge connections from the growth medium to two solution-filled glass vials, where the reference and counter electrodes were located. The three electrodes and their connection to a potentiostat completed the electrochemical cell (Fig. 1B). The setup was kept in a temperature-controlled incubator to maintain a consistent growth condition, with external stimulation controlled by the potentiostat.

To test the effect of distal phenazine oxidation on biofilm development, we used a *P. aeruginosa* phenazine-null (Δ*phz*) mutant [14] incubated on medium containing 100 ​μM phenazine methosulfate (PMS). *P. aeruginosa* produces at least five phenazines--phenazine-1-caboxylic acid (PCA), phenazine-1-carboxamide, 1-hydroxyphenazine, and the methylated derivatives 5-methyl-PCA (5-Me-PCA) and pyocyanin--that have different redox potentials and hydrophobicities. Although some of these phenazines have been demonstrated to act as electron shuttles [8], only the methylated phenazines--in particular 5-Me-PCA, which has the most oxidizing redox potential--prevent wrinkle formation. We previously established that the commercially available PMS is a good surrogate for 5-Me-PCA: supplementing agar with 200 ​μM PMS does not confer toxicity and is sufficient to prevent wrinkle formation in Δ*phz* colony biofilms [15,16]. For the current study, it was important to choose a PMS concentration that (i) is low enough to allow for colony wrinkling in the absence of electrochemical stimulation but also (ii) high enough for sufficient PMS-dependent electron transfer between the electrode and the colony biofilm. Both criteria were met by adding 100 ​μM PMS to the experimental setup.

For each experimental run, we compared two otherwise-identical setups: a “stimulated” case in which the potentiostat was used to generate an oxidative potential and an “unstimulated” case in which no potential was applied. The same two setups in the absence of PMS served to control for phenazine-independent side reactions. Time-lapse imaging was used to follow the development of biofilms over time. Percentage of biofilm wrinkle coverage was determined using a Laplacian-based edge-finding algorithm on the acquired images.

We found that applying a potential of +200 ​mV vs. Ag/AgCl was necessary to sufficiently oxidize bacterially reduced PMS and delay colony wrinkling (Supplementary Fig. 1). We observed a decline of redox peaks in cyclic voltammograms that were recorded for PMS in the growth medium before and after an experiment (Supplementary Fig. 2). A decrease has also been reported for other phenazines in long-term electrochemical setups and may partially be due to biofouling [8].

**Fig. 2:**
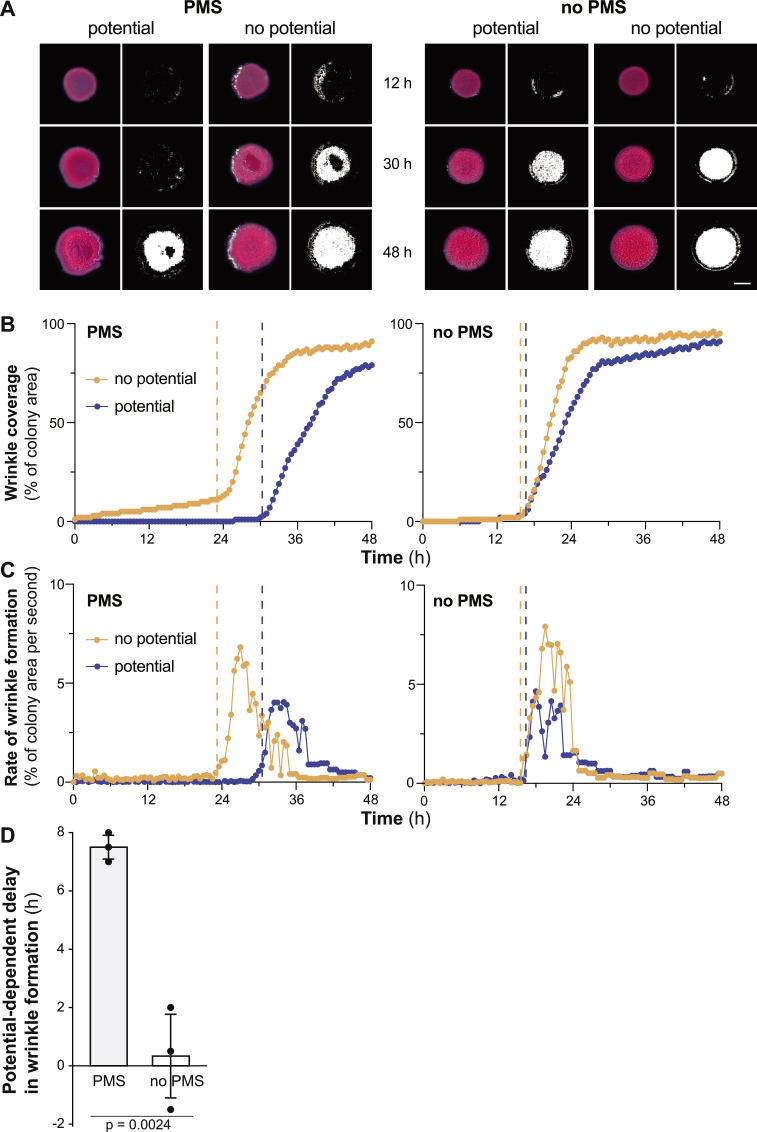
Effect of distal mediator oxidation on *P. aeruginosa* biofilm morphogenesis. Wrinkling was quantified by an algorithm described in the methods and representative data from experiments with and without PMS are shown. Panel **(A)** shows representative images of colony biofilms grown under each of the four experimental conditions as indicated. For each biofilm and time point, color images are shown on the left and black-and-white renderings from the wrinkle-detection software are shown on the right. Scale bar is 5 ​mm. Panels **(B)** and **(C)** show percent wrinkle coverage over time and rate of wrinkle formation, respectively, for colony biofilms shown in panel (A), incubated in the experimental system shown in Fig. 1B. Orange traces represent unstimulated biofilms, blue traces represent stimulated biofilms (i.e., those grown over an electrode poised at +200 ​mV). Dashed lines indicate the onset of wrinkling. Panel **(D)** shows the delay in onset of wrinkling for all experimental samples relative to unstimulated controls, in experiments conducted with (left) or without (right) PMS. Error bars represent standard deviation of three independent experiments. The p-value was determined using a two-tailed unpaired *t*-test. Stimulation had no appreciable effects on colony spreading (Supplementary Fig. 4).

Representative data for stimulated and unstimulated biofilms, incubated with or without PMS, are shown in Fig. 2A–C (see Supplementary Movie 1 for corresponding movies). Colony biofilms were first grown for 24 ​h without applying a potential. During this period, each colony grows thick enough to promote the formation of an O_2_ gradient such that it is undetectable at depth [12]. To then test whether electrochemically oxidizing PMS would delay wrinkle formation, we applied a potential of +200 ​mV vs. Ag/AgCl (where indicated) and began time-lapse imaging of the biofilms. Fig. 2A depicts a representative colony for each condition after 12, 30, and 48 ​h of stimulation. The amount and rate of wrinkle formation during the stimulation period are shown in Fig. 2B and C. Based on these graphs, we determined the time point for the onset of wrinkling. We note the PMS-dependent delay in onset of wrinkling when no potential was applied, which is in agreement with previous studies [15,16] and is due to the contribution of O_2_ to PMS oxidation. To assess the effect of an applied potential of +200 ​mV vs. Ag/AgCl we compared the delay in onset of wrinkling between stimulated and unstimulated colonies in the presence and absence of PMS. The data for a total of six experiments are shown in Fig. 2D and provided as Supplementary Dataset 1. Each experiment contained one stimulated and one unstimulated sample; three experiments were conducted with PMS and three without PMS to control for phenazine-independent effects of the applied potential (see Supplementary Fig. 3 for statistical analysis). In the presence of PMS, we found that stimulated biofilms showed a delay in the onset of wrinkle formation of 7.5 ​± ​0.4 ​h relative to unstimulated biofilms. This delay was diminished to 0.3 ​± ​1.4 ​h in control experiments where PMS was omitted from the medium (Fig. 2D). These results show that extracellular phenazine oxidation inhibits wrinkle formation in *P. aeruginosa* colony biofilms and confirm that phenazine redox state and/or the effects of phenazines on the cellular redox state are cues that control biofilm morphogenesis. Our observations also demonstrate that electron transfer to an electrode affects biofilm physiology and therefore have implications for mediated microbial fuel cells, which often employ biofilms as catalysts for current generation [[17], [18], [19], [20]]. They also raise the possibility that extracellular electron transfer could modulate biofilm development in other bacteria that reduce electron-shuttling compounds [[21], [22], [23], [24]].

Supplementary video related to this article can be found at https://doi.org/10.1016/j.bioflm.2020.100025

The following is the supplementary data related to this article:

Multimedia component 1 Multimedia component 1

## Materials and methods

### Strains and culture conditions

*Pseudomonas aeruginosa* strain UCBPP-PA14 Δ*phz* [14,25] was routinely grown in lysogeny broth (LB; 1% tryptone, 1% NaCl, 0.5% yeast extract) at 37 ​°C with shaking at 250 ​rpm.

Custom setups for biofilm growth with electrochemical stimulation were printed using a Stratasys Eden 260 VS 3D printer (CAD files are available at https://osf.io/jstey/). All components were soaked in ethanol, washed in a laboratory dishwasher, and UV sterilized before each use. For each experiment, two Petri-dish-like vessels (diameter: 35 ​mm; height: 10 ​mm) were each filled with ~9 ​mL colony morphology assay medium (1% tryptone, 1% agar (Teknova), 40 ​μM Congo red, 20 ​μM Coomassie blue, 10 ​μM KCl, with or without 100 ​μM PMS) containing a fully submerged RVC foam electrode (ERG Aerospace, 30 pores per inch). The medium was left to set for ~16 ​h at 25 ​°C and >95% relative humidity in the dark before inoculation. To prepare inocula for experiments, liquid cultures were inoculated from individual colonies on streak plates and grown for ~16 ​h. The cultures were diluted 1:100 and grown for 2.5 ​h to an optical density of 0.45–0.55 ​at 500 ​nm. These sub-cultures were spotted as three-microliter inocula onto the growth medium and left to dry for 10 ​min in a biosafety cabinet in the dark. Setups were then moved into growth chambers and incubated for 24 ​h. Where noted, one of the two identical setups was subsequently connected to a potentiostat for the application of the potential for an additional 48–72 ​h. All biofilms were grown at 25 ​°C and >95% relative humidity in the dark. All experiments, unless otherwise stated, were done in at least three independent replicates on different days.

### Electrochemistry

In “stimulated” samples, an oxidation potential was applied in the solidified colony morphology medium to control the redox-state of PMS. In order to maintain a stable oxidation potential for the duration of the experiment, Ag/AgCl (RE-5B, BASi, 206 ​mV vs. SHE) was used as the reference electrode material, submerged in a glass vial containing 3 ​M potassium chloride to minimize the reference potential during oxidative stimulation. A counter electrode made of the RVC foam submerged in 0.1 ​M KCl, and 10 ​mM sulfuric acid was used to balance the oxidative current from the working electrode. Solid-state salt bridges (0.1 ​M KCl, 1% agar) were used for electrical connections as well as diffusion barriers between the growth medium, the reference electrode, and the counter electrode.

The potentiostat (CHI760D, CH Instruments) controlled and monitored the oxidation process for up to 72 ​h. Unless otherwise stated, the working electrode was set at a constant +200 ​mV vs. Ag/AgCl (+406 ​mV vs. SHE) to oxidize the PMS ($E_{0}$ = ​+80 ​mV vs. SHE [26]) in the growth medium. A high overpotential was used to compensate for drifts that could arise from PMS adsorption to the electrode surface, liquid junction potentials, or IR drop within the electrochemical cell. Potential effects of side reactions are captured by the control experiment, for which the same potential was applied but PMS was not added to the growth medium.

### Time-lapse imaging

Still images were acquired every 30 ​min with Logitech 760E webcams mounted in the incubator. LEDs were controlled by an Arduino Mega 2560 and were turned on 3 ​s before, and turned off 1 ​s after, each image acquisition. This protocol maintained a consistent image quality within each experiment while minimizing light exposure on colony growth. All acquired pictures are 1920 X 1080 pixels in size and time-stamped for further processing.

### Image processing

Time-stamped pictures were processed using a set of Python scripts based on the OpenCV computer vision library, available at https://osf.io/jstey/. First, the portion of the image containing the colony was cut out from the full-sized image. Second, the colony image was converted to grayscale, processed with two-dimensional Laplacian operators, with the results binary thresholded for a black-and-white wrinkle detection. The threshold for a binary decision was determined manually for the respective lighting conditions, such that detection of colony features was optimized while that of artifacts was minimized. Lastly, colony location and radius were manually determined for 2–3 keyframes (radius at a certain time) in each time-lapse movie. We then derived the radius for each frame by linearly interpolating between the keyframes. We chose a radius that falls slightly short of the edge of the biofilm, where there is typically a strong reflection of the light source (LEDs). (This “glare” cannot be distinguished from regular wrinkling by our program, as the wrinkles are identified from the difference in light reflection from the surface roughness.)

## CRediT authorship contribution statement

**William Cole Cornell:** Conceptualization, Data curation, Formal analysis, Investigation, Methodology, Validation, Visualization, Writing - original draft, Writing - review & editing. **Yihan Zhang:** Conceptualization, Data curation, Formal analysis, Investigation, Methodology, Software, Supervision, Validation, Visualization, Writing - original draft, Writing - review & editing. **Anastasia Bendebury:** Conceptualization, Data curation, Formal analysis, Investigation, Methodology, Writing - review & editing. **Andreas J.W. Hartel:** Data curation, Formal analysis, Investigation, Validation, Visualization, Writing - review & editing. **Kenneth L. Shepard:** Conceptualization, Funding acquisition, Investigation, Project administration, Resources, Software, Supervision, Writing - original draft. **Lars E.P. Dietrich:** Conceptualization, Data curation, Formal analysis, Funding acquisition, Investigation, Methodology, Project administration, Resources, Supervision, Visualization, Writing - original draft, Writing - review & editing.

## Declaration of competing interest

The authors of this manuscript declare no conflict of interest.
