# Supplementary material for: Phenazine oxidation by a distal electrode modulates biofilm morphogenesis
Source: Biofilm. 2020 May 13;2:100025. doi: 10.1016/j.bioflm.2020.100025 (PMC7798475; doi:10.1016/j.bioflm.2020.100025)
Supplement: Multimedia component 2 [file mmc2.pdf]

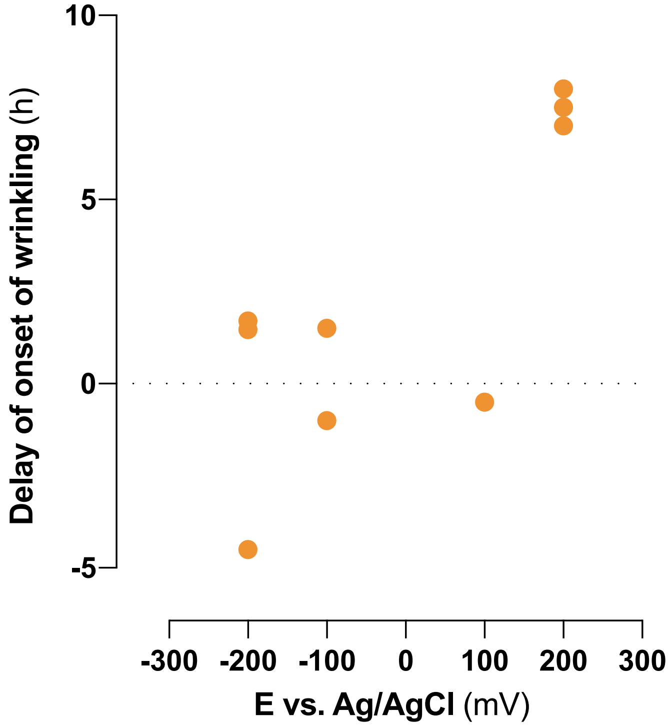

Supplementary Fig. 1. The effect of different applied potentials on the onset of colony wrinkling. We tested -200 mV (n=3), -100 mV (n=2), +100 mV (n=1), and +200 mV (n=3) vs. Ag/AgCl. An appreciable delay for the onset of wrinkling was observed only when we applied +200 mV vs. Ag/AgCl, indicating that this minimal potential is required to achieve sufficient oxidation of PMS in our e-chem setup.

Supplementary Figure 2

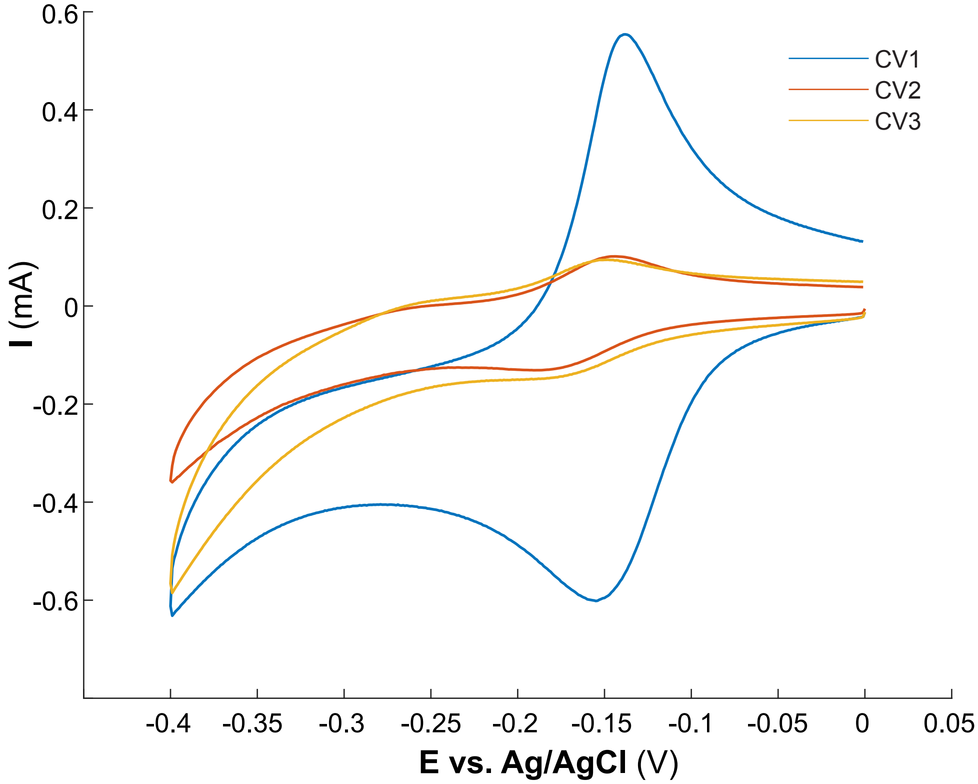

Supplementary Fig. 2. Representative cyclic voltammograms of PMS in our experimental setup (described in Figure 1). CV1 was taken at the start of an experimental run, before oxidative stimulation was applied. CV2 was taken at the end of an experimental run, after oxidative stimulation. CV3 was taken at the end of an experimental run in a control setup, which had not been subjected to stimulation. The difference in the peak heights between CV2 (0.041 mA, corresponds to a PMS concentration of 7.0 μM) and CV3 (0.068 mA, or 11.6 μM PMS) is much lower compared to their difference from CV1 (0.586 mA, or 100 μM PMS).

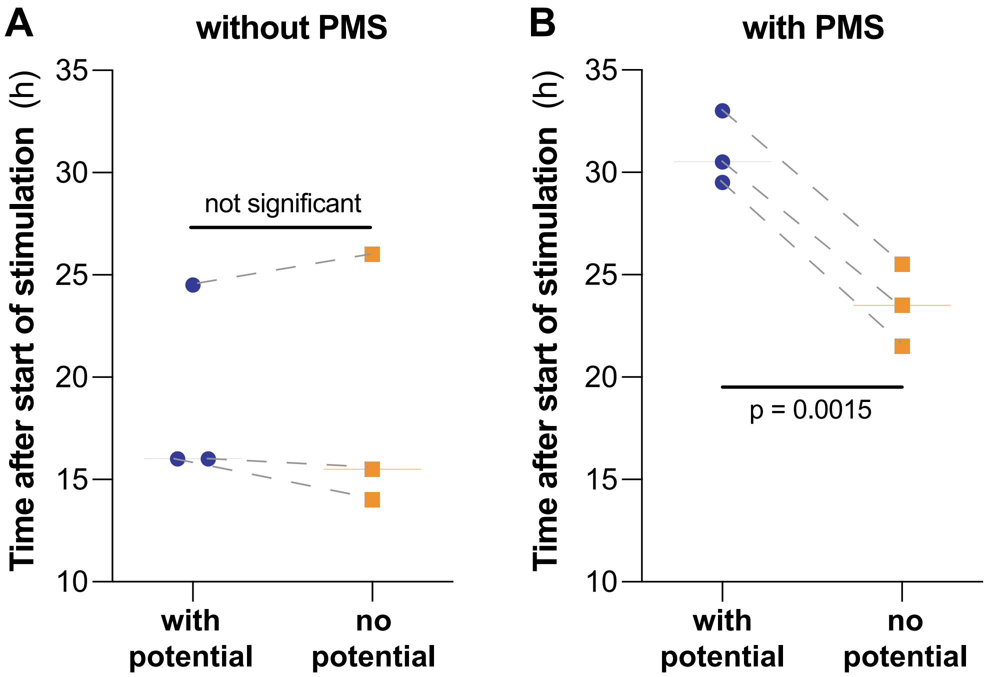

Supplementary Fig. 3. Effect of an applied potential on the onset of colony biofilm wrinkling in the absence **(A)** and presence **(B)** of PMS. Only in the presence of PMS does an applied potential of +200 mV vs. Ag/AgCl lead to a significant delay in the onset of wrinkling. The p-values were determined using a two-tailed paired t-test.

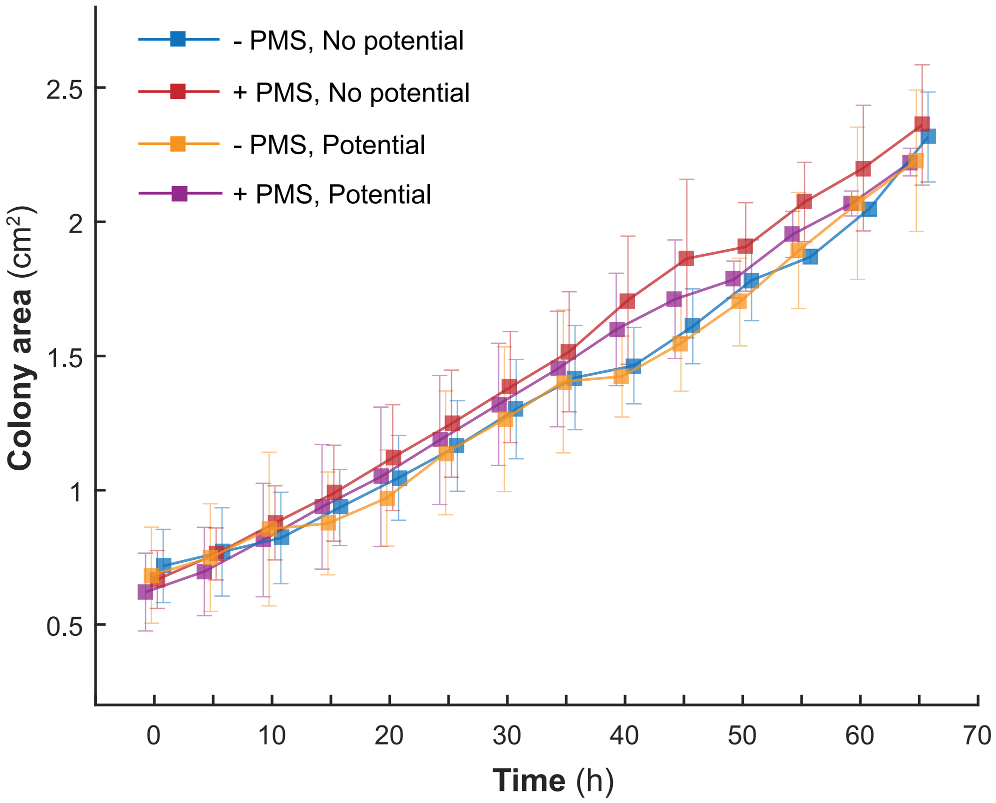

Supplementary Fig. S4. An applied potential of +200 mV vs. Ag/AgCl has no appreciable effect on colony spreading. Colony biofilm area was measured every 5 hours for 65 hours during the stimulation period. Biofilms were treated as described for Figure 2. Three biofilms for each of the four conditions were analyzed (with and without PMS; presence and absence of potential). Error bars represent standard deviation.
